# Disrupted brain mitochondrial morphology after in vivo hydrogen sulfide exposure

**DOI:** 10.1038/s41598-023-44807-y

**Published:** 2023-10-24

**Authors:** Wilson K. Rumbeiha, Dong-Suk Kim, Angela Min, Maya Nair, Cecilia Giulivi

**Affiliations:** 1 Department of Molecular Biosciences, School of Veterinary Medicine, University of California Davis, Davis, CA USA; 2 Medical Investigation of Neurodevelopmental Disorders (MIND) Institute UCDH, University of California Davis, Sacramento, CA USA

**Keywords:** Imaging, Cellular neuroscience

## Abstract

Changes in mitochondrial dynamics are often associated with dietary patterns, medical treatments, xenobiotics, and diseases. Toxic exposures to hydrogen sulfide (H_2_S) harm mitochondria by inhibiting Complex IV and via other mechanisms. However, changes in mitochondrial dynamics, including morphology following acute exposure to H_2_S, are not yet fully understood. This study followed mitochondrial morphology changes over time after a single acute LCt_50_ dose of H_2_S by examining electron microscopy thalami images of surviving mice. Our findings revealed that within the initial 48 h after H_2_S exposure, mitochondrial morphology was impaired by H_2_S, supported by the disruption and scarcity of the cristae, which are required to enhance the surface area for ATP production. At the 72-h mark point, a spectrum of morphological cellular changes was observed, and the disordered mitochondrial network, accompanied by the probable disruption of mitophagy, was tied to changes in mitochondrial shape. In summary, this study sheds light on how acute exposure to high levels of H_2_S triggers alterations in mitochondrial shape and structure as early as 24 h that become more evident at 72 h post-exposure. These findings underscore the impact of H_2_S on mitochondrial function and overall cellular health.

## Introduction

The brain orchestrates complex cognitive functions and vital physiological processes. Central to the brain's remarkable abilities are mitochondria, the cellular powerhouses responsible for generating the majority of adenosine triphosphate (ATP), the cell's energy currency. These organelles are crucial in maintaining cellular homeostasis, regulating apoptosis, and serving as a pivotal interface in the brain's response to various environmental stimuli. Hydrogen sulfide (H_2_S) is a common environmental toxicant and a crucial gaseous molecule naturally produced in the body^1^, including the brain ^2^.

The morphology and function of mitochondria in the brain have become subjects of increasing interest in scientific research. Recent investigations have revealed that mitochondrial dynamics and their structural adaptations can influence neuronal health, synaptic plasticity, and overall cognitive performance ^3–6^. Proper mitochondrial function is essential for sustaining neural activities, while its dysfunction may lead to neurodegenerative diseases and cognitive impairments ^7–9^. Mitochondria typically have an elongated shape, delimited by smooth outer and complex inner membranes ^10^. The tight and structured packing of the infoldings of the inner membrane (cristae) is functionally essential for increasing the surface area for ATP production^11–13^. The *intermembrane space*^14^ is located between the inner and outer membranes, whereas the *matrix* is the space inside the inner membrane. The latter subcompartment contains critical organic and inorganic compounds, biomolecules (mitochondrial DNA), soluble enzymes of the citric acid cycle, and structures (matrix granules, ribosomes) ^14^. Notably, the size (~ 0.5 to 3 µm), shape, and number of mitochondria vary considerably between cell types, developmental stage, age, and environmental factors, among others, and even within the chondriome^15^. The preservation of this dynamic homeostasis is critical for mitochondria to adapt to changing living conditions. As such, mitochondria in cells move, change shape, and fuse or split^16^ in a process called mitochondrial dynamics. This process is essential for maintaining their shape, distribution, and size; alterations of any of these are associated with numerous human diseases^17^.

In recent decades, the harmful effects of environmental pollutants on brain health have garnered considerable attention. One such environmental hazard is H_2_S. Once known solely for its toxic properties^18,19^, H_2_S has emerged as a remarkable gasotransmitter with versatile physiological functions in recent years^20–22^. H_2_S is now recognized as a key signaling molecule involved in diverse cellular processes, including vasodilation, inflammation, cognition, and neurotransmission. While the biochemistry and signaling roles of endogenous H_2_S continue to be extensively studied, its effects on cellular organelles, especially mitochondria, which are a well-known target for H_2_S-induced toxicity, have gained increasing attention. The intricate crosstalk between H_2_S and mitochondria has sparked considerable interest among researchers, as mounting evidence suggests that H_2_S may modulate mitochondrial morphology and function in a dose–response association, from signaling to toxic, detrimental health effects.

This study explored the fascinating interplay between a single acute H_2_S exposure and changes in brain mitochondrial morphology, shedding light on the potential implications for cellular health and disease. Only one other study indicated qualitative changes in mitochondria morphology in the brains of a rat model of alcoholism treated with aminooxyacetic acid (an inhibitor of cystathionine-beta-synthase) that increases endogenous H_2_S levels^23^, but not acute exposures. Here, we sought to elucidate the potential mechanisms through which H_2_S may influence mitochondrial dynamics in the brain. To this end, we analyzed 123 images obtained from the brains of mice exposed to 1000 ppm H_2_S for 30 min, and cohorts of surviving mice were sacrificed at 12, 24, 48, and 72 h post-exposure to generate a detailed picture of changes over time in mitochondrial morphology in murine thalamus. This dose was chosen to mimic acute H_2_S exposure following industrial accidents or nefarious acts ^24,25^. The thalamus was explored as it is one of the most sensitive brain regions upon H_2_S acute exposure ^19^. Understanding the complex interplay between mitochondria and H_2_S is essential for advancing our knowledge of neurobiology and environmental health, including neurodegeneration, and identifying potential therapeutic targets and strategies to mitigate the adverse effects of environmental pollutants on brain function. By shedding light on this critical intersection between cellular biology and environmental factors, this research may pave the way for novel interventions to safeguard brain health in an increasingly polluted world.

## Results

**Figure 1:**
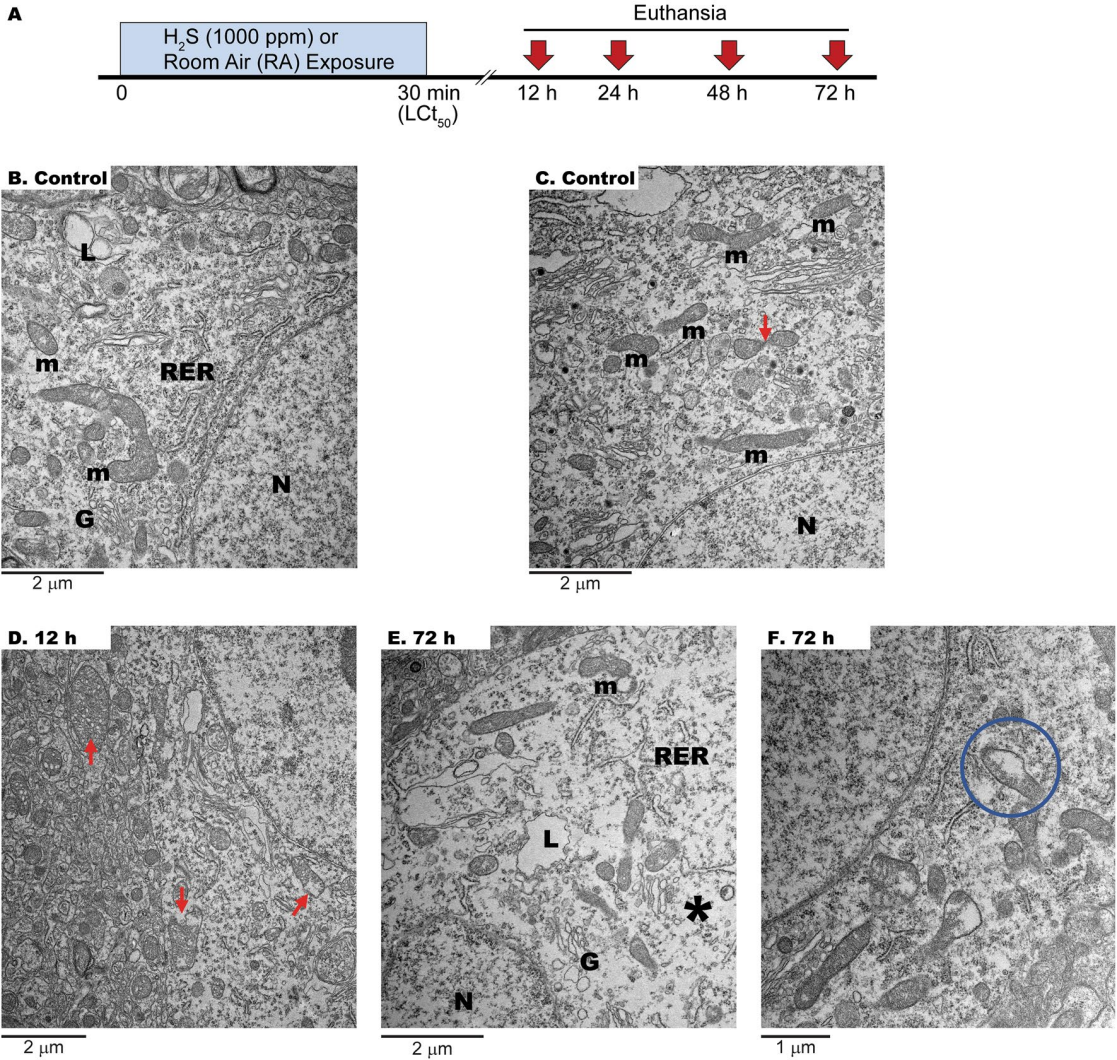
Experimental protocol and neuronal changes after acute H_2_S exposure. (**A**) A diagram summarizing the experimental procedure. Mice were exposed once to H_2_S at 1000 ppm for 30 min. Cohorts of surviving mice were euthanized at 12, 24, 48 and 72 h post-exposure. (**B,C**) Normal appearance of cytoplasm, organelles, and nucleus (N) in control brain samples from a mouse exposed to room air (normal mitochondria marked with m). (**D**) As early as 12 h after H_2_S exposure, mitochondria displayed swelling, dilation, and vacuolization (arrows). (**E,F**) At 72 h, significant cristae disruption was observed (circle marks magnification) with amorphous proteinaceous material, possibly resulting from unfolded, damaged proteins. The cytoplasm is rarefied (asterisk). *RER* rough endoplasmic reticulum, *m* mitochondria, *G* Golgi apparatus, *L* lysosomes.

Young adult C57BL/6 J male mice, aged 7–8 weeks, were randomly allocated to the (exposed) H_2_S group (*n* = 12) and the room air group (control; *n* = 4), following a protocol previously documented ^26,27^. The exposed mice were subjected to a single inhalation dose of H_2_S at 1000 ppm for 30 min, while the control group was exposed to room air through whole-body exposure. This H_2_S concentration was chosen because it replicates an acute H_2_S exposure scenario resembling accidental or intentional releases of this toxic gas ^24,28–30^. This exposure results in a 50% mortality rate, known as LCt_50_. Euthanasia of the surviving mice occurred at the following time points: 12, 24, 48, and 72 h post-exposure and their brains were promptly extracted and processed for electron microscopy to assess the impact of H_2_S on mitochondrial morphology (exposure paradigm shown in Fig. 1A). The focus on mitochondria was based not only on the role of this organelle in the CNS^31^, and the more potent inhibition of cytochrome *c* oxidase by H_2_S than cyanide^32^, but also because 50 mg/kg NaHS-treated mice showed a threefold and twofold increase in sulfides in myelin and mitochondria, respectively, compared to control animals^33^.

Representative images from transmission electron microscopy (TEM) of thalami from air-breathing animals show neuronal elements with well-preserved subcellular organelles (Fig. 1B, C). The rough endoplasmic reticulum (RER), and mitochondrial and cellular membranes are intact. The lysosomes and Golgi complexes are well preserved. Myelin sheaths show normal compaction with occasional loosening of myelin wraps. Nuclei show normal euchromatin. There is a scatter of electron-dense myeloid bodies in the neuropils.

In general, the inspection of the TEM images from H_2_S-exposed mice revealed a phenotype consistent with that of tissues affected by hypoxia, ischemia, and chemical hypoxia, akin to cyanide poisoning. At 12 h, mitochondria vacuolization, swelling, and cristae disruption are evident (Fig. 1D). Swollen mitochondria were identified by their expanded matrix space, fewer cristae, and less dense matrix staining. While these changes were observed as early as 12 h, those at 72 h were the most evident (Fig. 1D, F).

In some cases, the cristae were pushed to the side, and an amorphous material was present (likely unfolded, damaged proteins; Fig. 1F). Golgi complexes were swollen and disorganized (Fig. 1E). Lysosome and RER were enlarged following 72 h post-H_2_S exposure (Fig. 1E). At 72 h, neural soma cytoplasm showed rarefaction with aggregation of cytoplasmic contents and dilated endoplasmic reticulum (E.R; shown with asterisks; Fig. 1E). The neuropil contained axons, and neuronal nuclei are hypodense.

**Figure 2:**
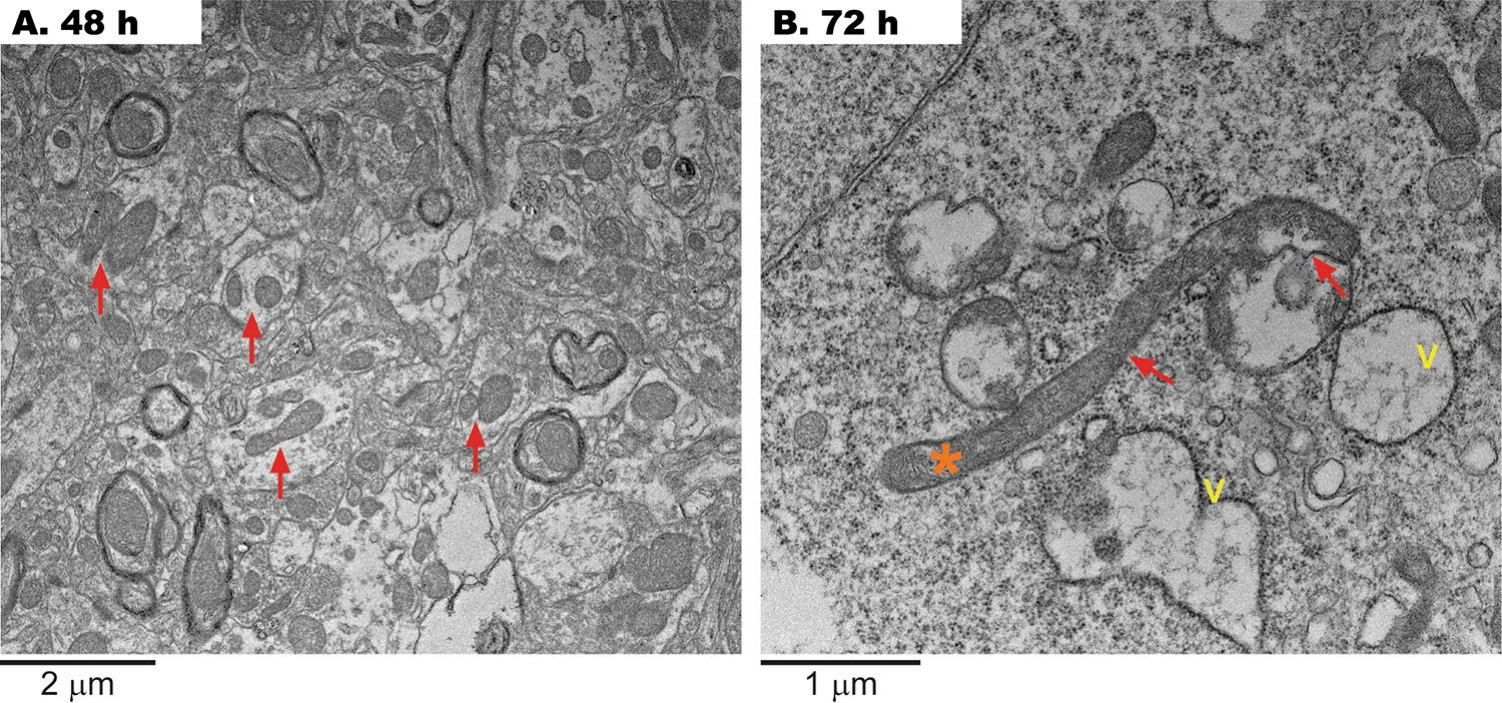
Late disruption of brain mitochondria fission after acute H_2_S exposure. (**A**) At 48 h, active fission was observed (arrows) to enhance mitophagy's clearance of damaged mitochondria. (**B**) At 72 h, the fission process seems halted, as judged by the appearance of long, damaged mitochondria (asterisk).

At 48 h, mitochondria fission was increased, possibly in an attempt to clear damaged mitochondria by mitophagy (Fig. 2A). However, this process seemed halted later with the presence of elongated, longer mitochondria at 72 h (Fig. 2B). Although these mitochondria have a larger size and elongation, they do not seem to be functional, as their cristae are sparse or loosely packed (Fig. 2B). Vacuolization of mitochondria was more evident at 72 h and observed as a loss of structured cristae, invaginations of the inner membrane, and matrix components accumulated to the side of the mitochondrial body (Fig. 2B).

**Figure 3:**
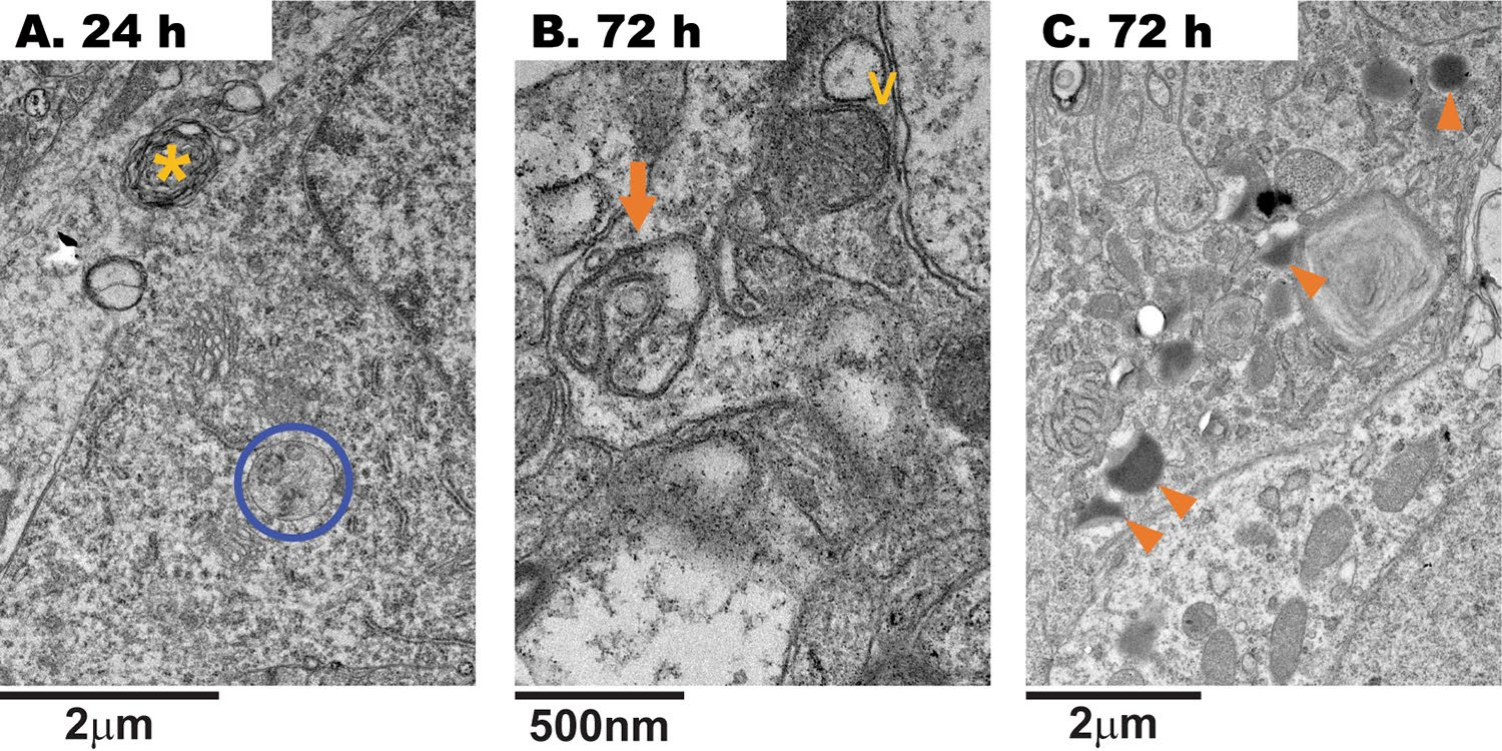
Generalized brain lesions after acute H_2_S exposure. (**A**) At 24-h after H_2_S exposure, a few double-membrane autophagosomes with engulfed organelles were observed (blue circle). (**B**) At 72-h, C-shaped double membrane structures (arrow) or double-membrane vacuoles (V) were observed. (**C**) At 72-h after H_2_S exposure, lipid droplets phagocytized by lysosomes were visible and stained dark (arrowheads). Both apoptotic and necrotic morphological features were seen (cell shrinkage, large chromatin clumps, damaged organelles, and deteriorated membranes).

At time points 48 h and 72 h, the degree of mitochondrial damage was more evident than at 12–24 h, accompanied by dilation of RER cisternae (loss and disruption of Golgi apparatus), loss of organized ribosomes on RER, large aggregates of smooth endoplasmic reticulum, presence of single-membrane vacuoles, and autophagosomes with lipid material (Fig. 3A–C).

At 24 h after H_2_S exposure, a few double-membrane autophagosomes with engulfed organelles were observed (Fig. 3A, blue circle). But at 72 h, C-shaped double membrane structures (arrow) or double-membrane vacuoles (V) were observed. Note at this time point, cytoplasm vacuolization was apparent (Fig. 3B), and lipid droplets phagocytized by lysosomes were visible and stained dark (arrowheads; Fig. 3C). Notably, lipid droplets are a well-documented source for the maintenance of mitochondrial membrane integrity and fatty acids for mitochondrial fatty acid β oxidation and have more recently been shown to play a protective role in the sequestration of toxic lipids that arise during autophagic degradation of membranous organelles^34^^.^

**Figure 4:**
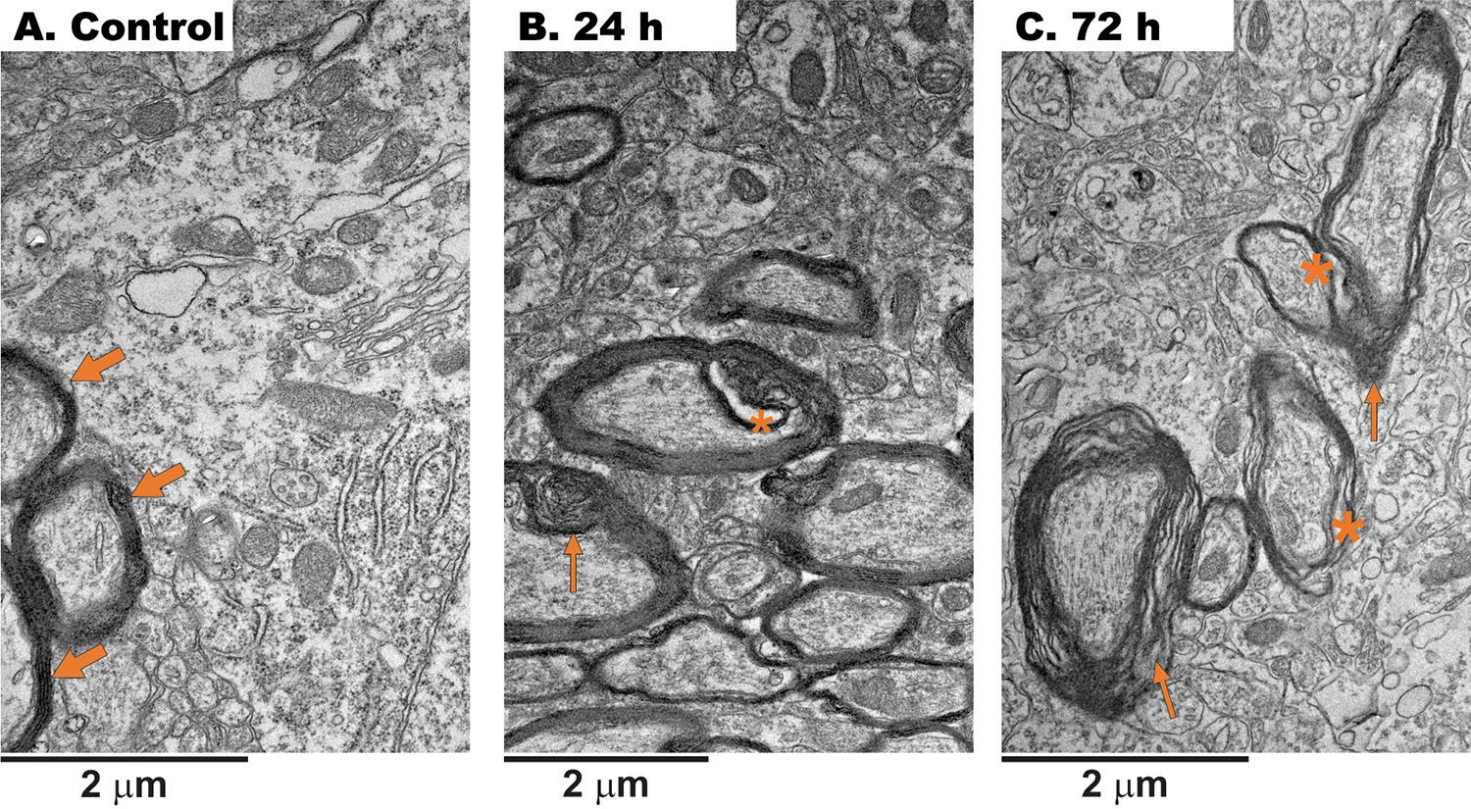
Myelin packing is affected by acute H_2_S exposure. (**A**) Under control conditions, myelin was well-developed, with a compact structure with axons enclosed in myelin visible (indicated by the arrows), and the layers exhibited a highly organized appearance. (**B,C**) In the H_2_S-exposed group, many small vacuoles (not quantified) were observed in the myelin at 24 h, 48 h, and 72 h (asterisks). In (**C**), myelin exhibited a loose structure and an increased inter-layer gap with visible stratification, which indicated a disrupted development (thin arrows).

We performed a qualitative analysis of TEM images on the myelin structure because relatively high sulfide content in myelin- and mitochondrial-enriched fractions was previously reported in mice dosed with 50 mg/kg NaHS^33^. Quantitative analysis of myelin thickness (for example ^35^) was not performed as it varies with neuron types within the brain region requiring specific staining for identifying the neuron type and biomarkers of myelin proteins. Under control conditions, myelin was well-developed, with a compact structure with axons enclosed in myelin visible (indicated by the arrows), and the layers exhibited a highly organized appearance (Fig. 4A). In the H_2_S-exposed group, many small vacuoles were observed in the myelin at 24 h (Fig. 4B), 48 h (not shown), and 72 h (asterisks; Fig. 4C). Myelin exhibited a loose structure and an increased inter-layer gap with visible stratification, which indicated a disrupted packing (thin arrows; Fig. 4C).

### Characterization of brain mitochondria after H_2_S exposure

**Figure 5:**
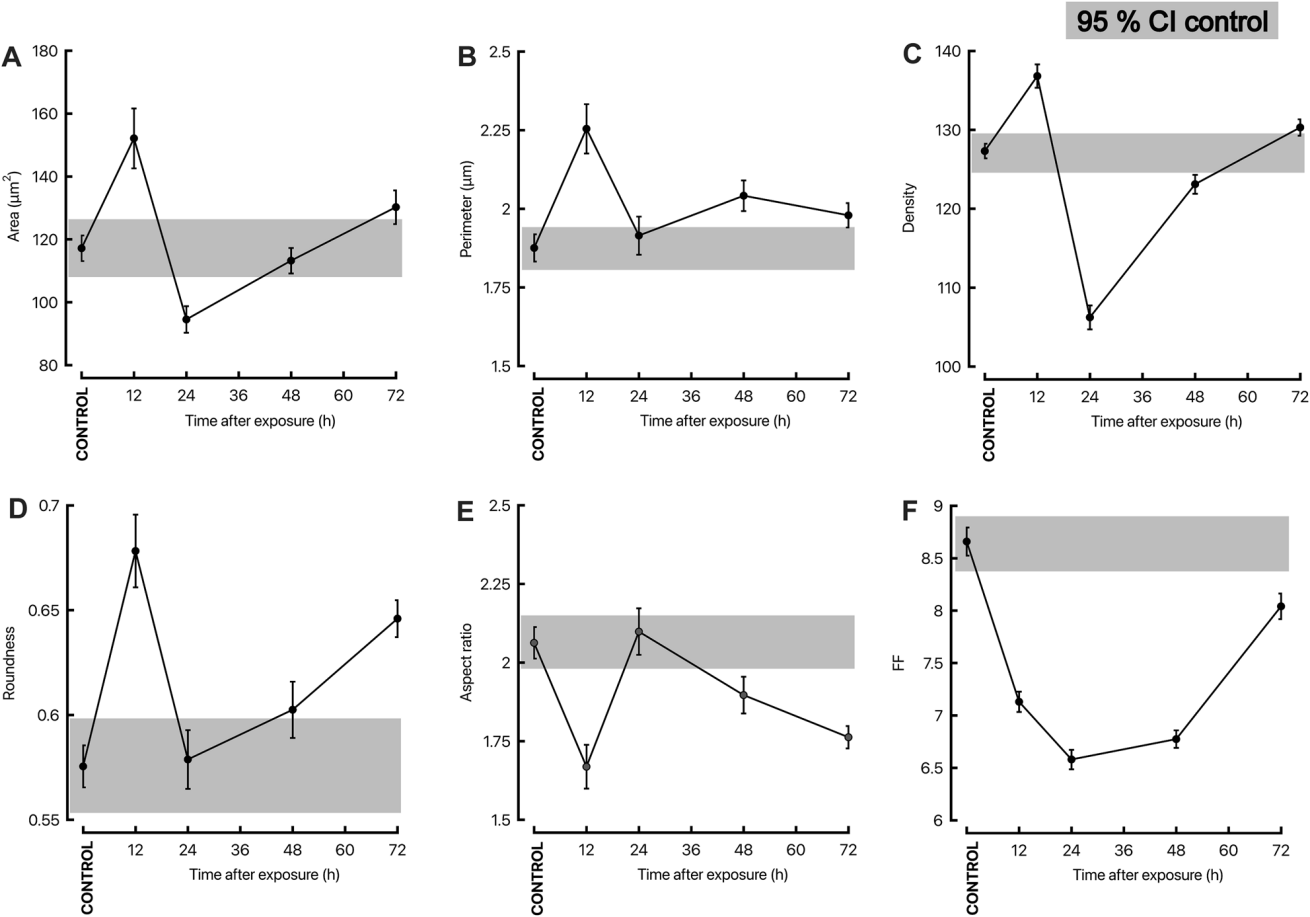
Acute hydrogen sulfide exposure alters mitochondria morphology. Parameters of mitochondrial morphology obtained with ImageJ software were the following: area (**A**), perimeter (**B**), density (**C**), roundness (**D**), aspect ratio (**E**), and form factor (**F**). Average values/conditions with S.D. are presented as a function of time for each of the five outcomes. The 95% confidence intervals built with control values from air-breathing animals are shown as grey rectangles, where the top border is the upper 95% CI limit, and the lower border is the lower 95% CI limit.

Using ImageJ software, quantitative measurements of morphometric parameters (surface area, perimeter, roundness, density, aspect ratio, and form factor) were performed on mitochondria from the TEM images (Fig. 5). Each panel contains the 95% confidence interval of control values (grey boxes). Values outside the 95%CI were considered abnormal. The area of mitochondria in the brains of H_2_S-exposed animals at 12 and 24 h post-exposure was higher and lower (respectively) than controls (air-breathing animals; Fig. 5A). At 72 h, the area of mitochondria from H_2_S-exposed mice was generally larger than that from control mice (Fig. 5A). A similar trend was observed for the perimeter (Fig. 5B). The density of mitochondria was higher at 12 h than control animals, followed by a significant dip at 24 h, and similar to the area outcome, reverting to almost control values by 72 h (Fig. 5C).

As the change from elongated to smaller, round mitochondria indicates fragmentation and cellular stress^36,37^, we assessed mitochondrial roundness as a surrogate for evaluating the extent of mitochondrial fragmentation. These data span from 0 to 1, with 1 being a perfect circle. Mitochondria from H_2_S-exposed animals at 12 h showed increased roundness compared to controls (Fig. 5D), whereas a steady increase in roundness was also observed at later time points. These results indicated a significantly increased early mitochondrial fragmentation at 12 h followed by a steady decrease in fragmentation at 48 h and later.

Finally, mitochondrial branching and network were assessed in the images. In response to stress, mitochondria can adapt in a short timeframe to increase ATP production (e.g., calcium-dependent activation of specific dehydrogenases within the citric acid cycle^38^). In contrast, long-term adaptation may include changes in the chondriome network, content, ultrastructure, and enzymatic levels^39,40^. As a decrease in mitochondrial network complexity can result from fragmented, rounder mitochondria and increased mitochondrial fission, we calculated the aspect ratio (Fig. 5E) and the form factor (Fig. 5F) to describe the mitochondrial network complexity^41,42^. Mitochondrial aspect ratio estimates length (i.e., the ratio between major and minor axes of an ellipse equivalent to the mitochondrion). The aspect ratio has a minimal value of 1, corresponding to a circular mitochondrion. The aspect ratio was significantly lower at 12 h (closer to a circle). In comparison to control, at 48 h and later, a trend towards increased roundness was noted (Fig. 5E). These results indicated increased round, circular mitochondria (and less elongated) in response to H_2_S exposure.

The form factor [form factor = 1/4 × (area/perimeter^2^)] is a number that combines measures of length and degree of branching^42^. Mitochondria with large aspect ratios are larger organelles, elongated, whereas those with high form factor scores contain more complex branching ^42^. As observed above, exposure to H_2_S resulted in a fall of the aspect ratio at 12 h, followed by a steady decline from 24 h and on (Fig. 5E). The form factor scores declined at all time points, with some recovery observed at 72 h, suggesting a disrupted chondriome network (Fig. 5F). These results indicated that exposure to H_2_S resulted in rounder mitochondria with altered cristae, likely associated with a disruption in the network.

**Figure 6:**
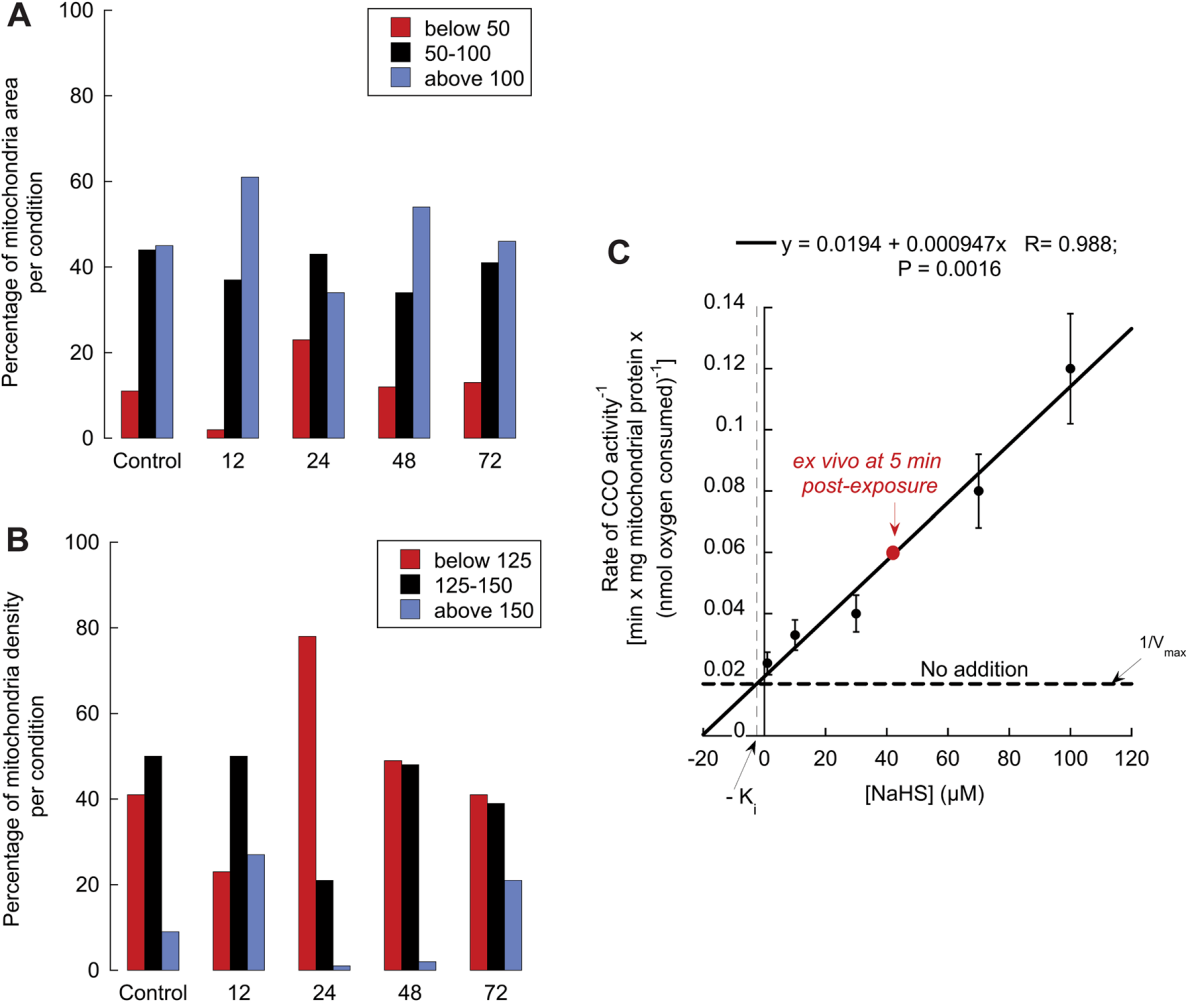
Effect of acute H_2_S exposure on the distribution of mitochondrial density and size and Complex IV activity. Values of density and area were obtained for each image and condition using ImageJ. The values were analyzed using JMP software (Supplementary Information). To visualize the effect of H_2_S on size (**A**) and density (**B**) distributions, range values with the most population from air-breathing animals (black) were taken as “controls”. Values below (red) or above (blue) this range were considered as smaller (size) (or less dense if density), or larger (size) (or denser if density). ImageJ assigns each pixel a value between 0 and 255 depending on the value/color. The integrated density is the sum of all pixel values inside a mitochondrion. It should also equal the (area in pixels)^2^ * mean pixel value (mean grey value). (**C**) Complex IV activity from rodent brains was evaluated by polarography as indicated in the “Methods”. As a H_2_S donor, NaHS was added at the indicated concentrations. Each experiment was done in triplicates, and the data are mean ± SD. The values were presented by using a Dixon plot and fitted to a linear regression (linear regression equation, R value, and Pearson’s P value shown in panel), in which the horizontal dashed line represents 1/V_max_ of Complex IV activity without any NaHS addition. The point at which the fitted line crosses the dashed one, and extrapolated to the X-axis, represents the Ki. The red point represents the ex vivo activity of Complex IV evaluated in brains of mice exposed to the same H_2_S paradigm as utilized in this study. Recalculated from ^26^.

Excessive mitochondrial fragmentation or ineffective clearance of damaged mitochondria after fission may generate smaller mitochondria. In addition, increases in dumbbell-shaped mitochondria can be explained by incomplete mitochondrial fission (halted or slowed in the final stages of this process) or an overall increase in the total number of mitochondria undergoing fission. Although the number of dumbbell-shaped mitochondria found in each image was insufficient for robust statistical analysis, the distribution of mitochondrial size and mitochondrial density aided the understanding of these options. To this end, we calculated the area distribution (as a surrogate for size; Fig. 6A) and density of mitochondria (Fig. 6B) of control and exposed animals. The values were binned into three categories (below and above the “control range”), considering most mitochondria in control animals as the “control range” (Supplementary Information). The population of smaller mitochondria increased at 24 h, whereas that of larger mitochondria was observed at 12 h and 48 h (Fig. 6A). The distribution of mitochondrial density (integrated density calculated as area × mean gray value of pixels for each mitochondrion) showed increases in the population with more density (likely clumped material) at 12 h (and 48 h and 72 h). A significant increase in the population of less dense mitochondria is observed at 24 h, likely due to vacuolization (Fig. 6B). Taken together, these results indicated that at 12 h, most mitochondria were larger and denser, whereas at 24 h, they were mostly smaller and less dense; at 48 h, they were larger than at 24 h with less density; at 72 h, they were of sizes comparable to controls but with more representation of denser mitochondria. These results suggested that the fission process of the damaged mitochondria at 12 h was not halted, resulting in smaller and “lighter” mitochondria at 24 h, increasing their sizes by 48 h, and at 72 h, resembling the distribution in size as those of controls but denser (similar to that at 12 h).

### Hydrogen sulfide exposure affects morphology and mitochondrial activity

Numerous works ^18,27,43–58^, including publications from Dr. Rumbeiha’s team^26^ have shown decreased cytochrome *c* oxidase activity following acute H_2_S exposure of animals. Inhibition of cytochrome *c* oxidase activity reduces ATP production as it is the terminal oxidase of the electron transport chain. Complex IV activity was evaluated in situ in isolated and purified brain mitochondria from mice by using the artificial substrates ascorbate/TMPD in the presence of the Complex III inhibitor antimycin with various concentrations of NaHS as a hydrogen sulfide donor. The range of doses tested was up to 100 µM which includes the equivalent of brain sulfide after acute exposure to 1,000 ppm H_2_S gas, which is approximately equivalent to 15 mg NaHS/kg i.p. (270 µM NaHS in the bloodstream assuming homogenous distribution; recalculated from ^33^) and taking into account that at the physiological pH of 7.4, approximately one-third of the sulfide, whether derived from gaseous H_2_S or one of its alkali salts, will exist in the form of H_2_S (Fig. 6C). The data were presented as a Dixon plot (NaHS concentration vs. reciprocal of the Complex IV activity) because it evaluates whether the inhibitor concentration follows a linear correlation with the reciprocal of the velocity of the activity and the calculation of the Ki. The calculated Ki (2.5 µM) was similar to that obtained at pH 8.05 (2.6 µM^59^), consistent with the more basic pH of the mitochondrial matrix (pH ~ 8.0^60,61^) and with the increase in inhibition efficiency with decreasing pH^59^. Thus, an endogenous donor of H_2_S at concentrations equivalent to those of an acute exposure inhibited Complex IV activity by 83%, indicating that lower ATP production by mitochondria likely accompanied the changes in morphology. We also recalculated brain Complex IV activity evaluated ex vivo from an identical in vivo, whole animal exposure paradigm^26^ (Fig. 6C, red round marker). The inhibition of the activity seemed underestimated because it corresponded to a concentration of NaHS of about 40 µM vs. the expected one of ~ 100 µM, suggesting that the degree of inhibition by H_2_S of Complex IV in vivo could be substantially higher, especially when samples are extracted under ambient air from animals exposed to H_2_S gas and later stored at −80 °C for relatively long times. However, we cannot exclude that the inhibition reported by such studies may reflect other damaging effects on this Complex that do not include the competitive inhibition of this gas on Complex IV activity.

### Mitochondrial morphology scores after hydrogen sulfide exposure

**Figure 7:**
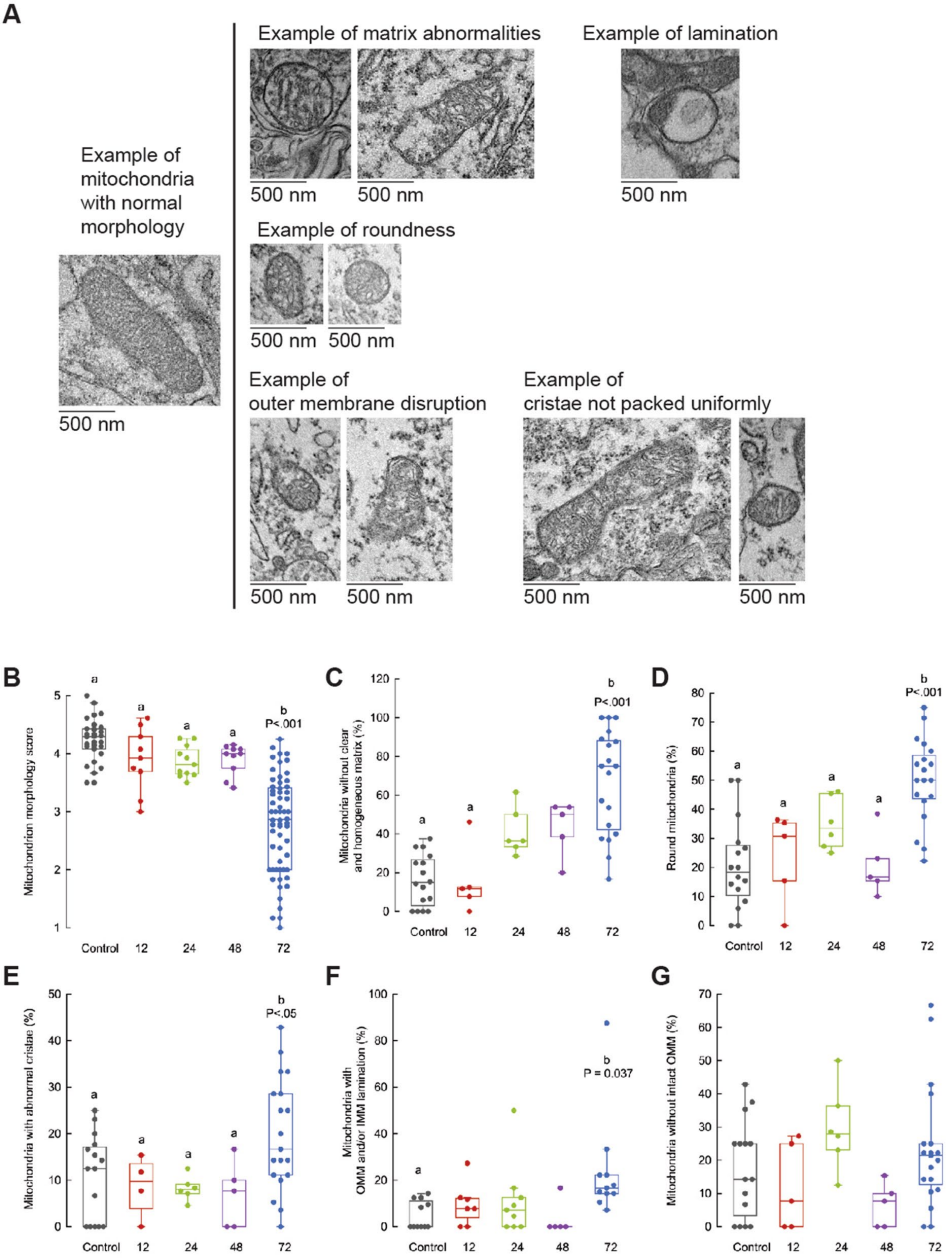
Quantitative comparison of a range of mitochondrial morphometric outcomes obtained after acute H_2_S exposure. Top panel. Example of mitochondrial images with disrupted morphology after acute H_2_S exposure. Representative images were selected to show the breadth of disrupted mitochondrial morphology assessed in this study. Full details are provided in the main text. Bottom panel. Each data point represents the individual value of a mitochondrion within each condition. Box and whisker plots were used to visualize the spread of data points under each condition. Each box (interquartile distance) encloses 50% of the data, with the median value of the variable displayed as a line. The top and bottom of the box mark the limits of the ± 25% of the variable population (i.e., upper and lower quartiles). The lines extending from the top and bottom of the box (whiskers) mark the minimum and maximum values within the dataset that fall within an acceptable range. Any value outside this range, called an outlier, is displayed as an individual point. Mitochondrion morphology score (**A**) spanning from 1(worst) to 5 (best). (**B–F**) The percentage of mitochondria with a particular feature under each condition [abnormal mitochondrial matrix (**B**), increased roundness (**C**), abnormal cristae (**D**), increased lamination (**E**), and abnormal outer mitochondrial membrane (**F**)]. ANOVA tests were run for each parameter, followed by Tukey's post-hoc. Equal letters mean no statistical differences.

To study the classification of mitochondria based on five criteria relative to the control air-breathing mice, we randomly selected 50% of the images/group as representative examples for further detailed structural analyses (Fig. 7). We systematically checked on the following criteria: (1) mitochondrial matrix appearance; (2) mitochondrion shape (round vs. oblong); (3) cristae morphology; (4) intactness of mitochondrial outer membrane; and (5) presence of separation between mitochondrial inner and outer membranes (or lamination). The presence of a negative (detrimental) outcome was considered as zero, whereas a positive (beneficial) one was evaluated as a 1. The sum of these individual scores allowed for obtaining an overall mitochondrial morphology score (from 1, the worst, to 5, the best). These scores were obtained for all mitochondria within each image and averaged per image. Examples of the different aspects of this scoring system are shown in Fig. 7, top panel.

Figure 7 bottom panels summarize the resulting mitochondrial morphology scores obtained at each time point. At 72 h, most mitochondria showed a 34% decrease in the overall mitochondria morphology scores compared to controls (worse; mean ± SEM: 2.8 ± 0.1) than those at all previous time points (for control, 12 h, 24 h, and 48 h, respectively: 4.23 ± 0.07; 3.9 ± 0.2; 3.87 ± 0.08; 3.91 ± 0.08; Fig. 7A).

As the overall score was based on the sum of the scores of the 5 criteria indicated above, we explored whether any particular feature weighted the most towards the lower scores observed at 72 h. This analysis (based on the percentage of mitochondria with a specific feature/image) was relevant as it can provide information on the time course of H_2_S’s effect on mitochondrial morphology.

### Criterion 1, mitochondrial matrix appearance

This criterion allowed us to assess abnormal accumulations of calcium deposits, clumps, electron-lucent vacuoles, enlarged electron-dense matrix granules, and lipid material in the mitochondrial matrix, indicating impaired quality control mechanisms. Healthy mitochondria rely on efficient mechanisms for removing damaged components through processes like mitophagy, and dysfunctional mitochondria with excessive calcium may evade proper degradation or indicate cellular stress. At 72 h, most (~ 80%) mitochondria presented enlarged matrix granules, filamentous intramitochondrial crystalline inclusions, and clumped, dense materials compared to controls or those at 12 h after the exposure (< 20%; Fig. 7B), suggesting a decreased space for normal activities of the citric acid cycle and anaplerotic reactions.

### Criterion 2, mitochondrial shape

Assessing mitochondria shape (oblong vs. round) allowed for identifying issues with mitochondria dynamics (balance between fission–fusion processes). Imbalances in these processes may lead to mitochondrial dysfunction and have been linked to various diseases, including neurodegenerative disorders. In addition, and as indicated above, changes in mitochondrial morphology, such as elongation (oblong mitochondria) or fragmentation (round mitochondria), could suggest cellular stress and damage. At 72 h, about half of the mitochondria had a round shape compared to those at previous time points (from 18 to 32%; Fig. 7C). Relevant to the brain, the morphology of mitochondria in neurons is closely linked to synaptic function and plasticity. Healthy neurons often have elongated mitochondria that can efficiently move to the synapses to supply energy and calcium buffering. The increase in round mitochondria in neurons after H_2_S exposure may indicate impaired axonal transport or dysfunction. Furthermore, mitochondrial shape and size changes are tightly linked to the process of mitophagy, where damaged or dysfunctional mitochondria are selectively removed. The increase in round mitochondria at 72 h post-exposure may reflect deficiencies in the rates of mitochondrial turnover and mitophagy, essential for maintaining cellular homeostasis.

### Criterion 3, cristae morphology

The relevance of assessing cristae morphology relies on evaluating the extensive surface area that provides for the electron transport chain (ETC) and oxidative phosphorylation. These processes are central to synthesizing energy-rich ATP molecules. As such, cristae's organization and morphology directly impact these energy-producing processes' efficiency. Packing cristae and the proximity of components within the ETC facilitate efficient electron transfer and minimize energy loss during mitochondrial respiration. Any cristae morphology and packing alterations can affect the ETC's function, leading to reduced ATP production and increased reactive oxygen species (ROS) generation. Cristae structure also influences the mitochondrial membrane potential, essential for maintaining proper mitochondrial function and regulating cell death pathways.

As observed before, at 72 h post-exposure, a significant number of mitochondria showed disorganized, disrupted cristae, with some showing complete loss of regularly arranged cristae throughout the entirety of the mitochondrial matrix (Fig. 7D). The increased cristae structure disruption after H_2_S exposure may point to disruptions in the membrane potential, potentially leading to apoptosis or cellular dysfunction. In addition, as the shape and arrangement of cristae are closely related to mitochondrial dynamics, including fission and fusion events, altered cristae morphology may impact this process, affecting mitochondrial distribution, turnover, and response to cellular stress.

### Criterion 4, separation of outer and inner membranes (lamination)

Under this criterion, we recorded mitochondria where the outer and inner membranes were abnormally separated. Many mitochondria at 72 h post-exposure showed displaced cristae with a clear lack of association between inner and outer membranes and an absence of concentric distribution (Fig. 7E). Usually, this feature involves the presence of vacuolization. Similar changes had been observed in other experimental models, such as hypoxia-ischemia^62,63^. This is consistent with an initial inhibition of oxidative phosphorylation (OXPHOS), followed by abnormal protein synthesis (enlargement of the endoplasmic reticulum).

### Criterion 5, intactness of mitochondrial outer membrane

An intact outer membrane is crucial for maintaining the structural integrity of mitochondria as this biological barrier separates the mitochondria from the cytosol, creating a specialized environment for mitochondrial functions and allowing for controlled and compartmentalized biochemical processes within the organelle. In addition, the outer membrane contains various transporters and channels that facilitate the transport of metabolites, ions, and other molecules between the cytosol and mitochondria. The outer membrane also houses the protein import machinery, facilitating nuclear-encoded proteins' transport into the mitochondrial matrix. It also involves signaling and communication between mitochondria and other cellular compartments. For instance, interactions with the endoplasmic reticulum are critical for calcium homeostasis and lipid metabolism. When the outer membrane becomes compromised, it can lead to mitochondrial fragmentation, disrupting mitochondrial dynamics and function and initiating mitophagy. No significant differences were observed in the intactness of the mitochondrial outer membrane across time points after H_2_S exposure (Fig. 7F).

## Discussion

This study focused on determining the impact of a single acute H_2_S exposure on mitochondrial morphology from rodent thalami. The analyses evaluated transmission electron microscope images from mice euthanized at 12, 24, 48, and 72 h after a single acute exposure of 1000 ppm H_2_S. This dose was chosen to mimic acute H_2_S exposure following industrial accidents or nefarious acts ^24,25,28–30^. Our analysis indicated a rapid metabolic crisis in thalami, evidenced by dramatic changes in mitochondrial morphology ensuing from a single acute exposure to H_2_S. The shift in mitochondrial distribution to smaller sizes and the loss of mitochondrial complexity, including cristae, provide solid evidence for injured mitochondria with reduced ATP production. The data on Complex IV activity performed with a H_2_S donor at concentrations equivalent to the in vivo acute exposure confirmed that hydrogen sulfide exposure (at least at short times after the exposure) is accompanied by morphological changes.

From the morphological assessment, we inferred that the energy-producing capacity of mitochondria, particularly concerning their normal-sized counterparts, is compromised, evidenced by the disorganization and insufficient presence of the inner mitochondrial membrane, where the well-established infoldings known as cristae are found. These cristae play a vital role by increasing the surface area available for ATP production, a key process in cellular energy generation. Upon close examination, it becomes evident that the network within these mitochondria is in disarray, further supporting our conclusion of reduced energy-producing capabilities. The disruption in the organization of the mitochondria likely extends to their ability to undergo mitophagy—the process of recycling and clearing damaged mitochondria from the cell. This disruption in mitophagy can be attributed to changes in the shape of the mitochondria, indicating potential challenges in their clearance and turnover.

While the smaller, rounder mitochondria at early time points may seem targeted for mitophagy, the actual decline in aspect ratio, vacuolization, disrupted matrix, and abnormal cristae at 72 h after H_2_S exposure, point to a lingering, halted mitophagy possibly arising from a defective fission–fusion balance. It is known that stress derived from starvation induces an increase in fusion activity resulting in elongated mitochondria to increase the efficiency of the adenosine 5′-triphosphate (ATP) synthesis and protect mitochondria from autophagosomal degradation, thereby enhancing cellular survival during nutrient deprivation^64^. However, if H_2_S were to induce elongation or enhance the fusion of mitochondria to escape autophagosomal degradation and enhance ATP production, they should show an increased, intact, and packed cristae structure^65^; however, these elongated mitochondria show cristae sparsely or unevenly arranged (Figs. 2B, 5, 6A, B) suggesting that their presence is not the result of an attempt to compensate the energy deficit or mitophagy. The larger size may be ascribed to significant mitochondrial swelling which occurs only after the loss of mitochondrial membrane potential (Δ*Ψ*_m_) and long after the release of cytochrome *c*^66^*.* Taking into consideration the disruption of the matrix and cristae structures and the lack of statistically significant outer membrane damage, it is tempting to propose that the early origins of disrupted mitochondria morphology upon acute H_2_S exposure reside at the matrix and inner membrane consistent with the early inhibition of Complex IV by H_2_S ^50^ and increased oxidative stress-mediated damage ^67,68^.

Results also show that acute H_2_S exposure significantly injured neuronal myelin sheaths, and endoplasmic reticulum. Myelin sheaths were split and edematous, and axons were rarified and dilated. These neuronal changes corresponded to time course changes noted in mitochondrial morphology. Mitochondrial swelling was notable at the earliest observation time (12 h), and injury was even more evident at 72 h. These results suggest that a single acute exposure to H_2_S triggers a cascade of events that lead to mitochondrial injury. As mitochondrial damage worsened (72 h), so did injury of other neuronal cellular components, including the endoplasmic reticulum, Golgi complex, lysosomes, and myeline sheaths (Figs. 1D, E, 4B, C).

Limitations of this study included (i) the use of male mice only, which precluded the evaluation of the sex effect on H_2_S intoxication as others have reported^45,69^, (ii) the analysis performed on surviving mice which may bias the results assuming unperceived advantages to acute exposure, and (iii) the putative extrapolation to humans considering species differences^70^ and similarities^71,72^ including the use of 7–8 weeks old mice (considered as young adults) whose maturational rate does not linearly correlate with humans. Finally, the substrain C57BL/6J has negligible mitochondrial nicotinamide transhydrogenase activity due to a deletion comprising the *Nnt* gene^73^, and as such, an increased background of mitochondrial oxidative stress^73,74^, which may have resulted in the presentation of a more severe phenotype.

In conclusion, our findings show that acute exposure to 1000 ppm of H_2_S for 30 min (LCt_50_) directly impacts the structural integrity and functional efficiency of mitochondria from thalami. This is primarily manifested through the disorganization and scarcity of the inner mitochondrial membrane. Furthermore, the altered network organization and compromised mitophagy accentuate the challenges these mitochondria face in maintaining optimal energy production and cellular health. This comprehensive exploration of the interactions between H_2_S exposure and mitochondrial morphology aims to contribute to the growing knowledge surrounding the intricate crosstalk between friend H_2_S gasotransmitter and foe H_2_S xenobiotic on cellular organelles. The findings from this study may pave the way for novel therapeutic strategies harnessing the potential of H_2_S to modulate mitochondrial function (e.g., activate mitophagy), thus offering new avenues for improving overall cellular health.

## Materials and methods

### Animals

All animal studies were approved by the Institutional Animal Care and Use Committee (IACUC-18-136, Feb. 2019) of Iowa State University (ISU). This study is fully aligned with the ARRIVE 2.0 guidelines^75^, ensuring transparent and comprehensive reporting of the research methods utilized in this study and its outcomes. Adhering to these guidelines enhances research quality and reproducibility, underscoring our commitment to upholding rigorous standards and promoting transparency in our findings. We confirm that all experiments were performed in accordance with relevant guidelines and regulations. Animals were treated humanely and handled with care. The 7–8-week-old male C57BL/6J mice were purchased from the Jackson Laboratories (Bar Harbor, ME). Mice were housed in five per cage in the laboratory animal resources (LAR) at the College of Veterinary Medicine, ISU, with a 12:12 h light and dark cycle. Room temperature and relative humidity were maintained at 22 ℃ and 35–50%, respectively. Protein Rodent maintenance diet (Teklad HSD Inc., WI) and drinking water were provided ad libitum. Mice were acclimated for at least 72 h before the start of the experiments.

### Exposure paradigm

C57BL/6J male mice 7–8 weeks old (considered young adults) were randomly assigned to each group (*n* = 12 for the H_2_S-breathing group and *n* = 4 for the control or ambient air-breathing group), following a protocol published before ^26,27^. The experimental protocol is summarized in Fig. 1A. Mice were exposed to a single dose of H_2_S at 1000 ppm (exposed) or room air (control) by inhalation (whole-body exposure) for 30 min. This exposure causes 50% mortality (LCt_50_), resulting in 3–4 surviving mice/group (except the control). Male mice were used because previous work by W.K.R. showed that they are more sensitive to acute H_2_S exposure than females^27^. High purity of H_2_S gas (> 99.9%) or room air (> 98%) were supplied from gas cylinders (Airgas, IA). The concentration of H_2_S in the exposure chamber was monitored in real-time using a H_2_S sensor (RKI Instruments, Union City, CA). Mice were euthanized at 12, 24, 48, and 72 h after the single acute exposure to assess the time course effects of H_2_S on mitochondrial morphology. The dose of 1000 ppm was chosen to mimic acute H_2_S exposure following industrial accidents or nefarious acts. Only mice surviving acute exposure were used in this study. Mice were euthanized at each time point by decapitation using a guillotine. Mouse brains were immediately removed. The thalamus was micro-dissected on ice, blocked in 1% paraformaldehyde, 3% glutaraldehyde in 0.1 M sodium cacodylate buffer, pH 7.2 at 4 °C, and processed for electron microscopy.

### Electron microscopy

Micro-dissected thalamus brain tissues were placed into 1% paraformaldehyde, 3% glutaraldehyde in 0.1 M sodium cacodylate buffer, pH 7.2, and fixed for 48 h at 4 °C. The thalamus tissues were washed in the cacodylate buffer for 10 min three times and post-fixed with 1% osmium tetroxide in 0.1 M sodium cacodylate buffer for one h at 20–22 °C. Subsequently, the thalami were washed thrice with deionized water for 15 min each. The thalamus tissues were washed in distilled water for 10 min and dehydrated through a graded ethanol series (25, 50, 70, 85, 95, 100%) for 1 h each step. This was followed by dehydration, with three changes of pure acetone, 15 min each, and infiltrated with EmBed 812 formula (hard) for EPON epoxy resin (Electron Microscopy Sciences, Hatfield PA) with graded ratios of resin to acetone until thoroughly infiltrated with pure epoxy resin (3:1, 1:1, 1:3, pure) for 6–12 h per step. Thalami were placed into BEEM^®^ embedding capsules and polymerized at 70 ℃ for 48 h. Thick sections (1.5 μm) were made using a Leica UC6 ultramicrotome (Leica Microsystems, Buffalo Grove, IL) and stained with EMS Epoxy stain (a blend of toluidine blue-O and basic fuchsin). Thin sections were made at 50 nm and collected onto single-slot carbon film grids. TEM images were gathered using a 200 kV JEOL JSM 2100 scanning transmission electron microscope (Japan Electron Optics Laboratories, USA, Peabody, MA) with a GATAN One View 4 K camera (Gatan Inc., Pleasanton, CA).

### Overall assessment of the brain chondriome and other cellular structures after H_2_S exposure

To evaluate the effect of hydrogen sulfide on brain mitochondrial morphology, we assessed 123 images from C57BL/6J male mice, of which 94 were from those mice exposed to H_2_S. The observations reported in this study represent approximately 30 min/image for 3900 h performed blindly by three independent reviews and over 400 h of subsequent data analyses.

### Morphometry and statistical analyses

Quantitative measurements of a range of common morphometric parameters were performed on individual mitochondria using ImageJ. Object measures, including surface area, were obtained using the methods described before^76^. ANOVA tests followed by Tukey's post-hoc were performed assuming equal or unequal variance based on the F-score, with the level of significance set at 0.05. Statistical analysis was performed using GraphPad Prism (version 7.02).

### Effect of NaHS on mitochondrial complex IV activity

Mice in a C57BL/6NJ background were kept in facilities approved by the Association for Assessment and Accreditation of Laboratory Animal Care International (AALAC). The animals were housed in Plexiglas cages (2–4 animals per cage; 55 × 33 × 19) and maintained under standard laboratory conditions (21 ± 2 °C; 55 ± 5% humidity) on a 12 h light/dark cycle, with ad libitum access to both water and food. The mice were fed with a standard rodent chow. All animals were handled by protocols approved by the University of California at Davis Institutional Animal Care and Use Committee. All protocols using animals followed the "Principles of laboratory animal care" (NIH publication No. 86-23, revised 1985). To isolate mitochondria from brains, mice were humanely euthanized by CO_2_ inhalation.

All reagents used for this study were of analytical grade or higher. Brain mitochondria from mice (2–3 months old) were utilized for testing the effect of hydrogen sulfide on Complex IV. After euthanasia, whole brains were dissected from the skull and placed in ice-cold PBS buffer to remove excess of blood. Subsequently, mitochondria were isolated by differential centrifugation as described before^77^. Protein was determined by using the BCA method from Pierce.

Mitochondria were evaluated by polarography under phosphorylating and non-phosphorylating conditions with NADH- and FADH_2_-linked substrates. Briefly, oxygen consumption was evaluated using a Clark-type oxygen electrode (Hansatech, King’s Lynn, UK) as described ^31^. Mitochondria (10–20 µg protein) were added to the oxygen chamber in a buffer containing 0.22 M sucrose, 50 mM KCl, 1 mM EDTA, 10 mM KH_2_PO_4_, and 10 mM HEPES, pH 7.4. To ensure the quality of these preparations, the oxygen consumption rates were evaluated in the presence of (i) 1 mM malate-10 mM glutamate (State 4) followed by the addition of 1 mM ADP (State 3) or with (ii) 10 mM succinate (State 4) followed by the addition of 1 mM ADP (State 3). This biological preparation’s respiratory control ratios (RCR = State 3/State 4) were 5 or above regardless of the substrate, indicating a high coupling between electron transport and ATP production. In situ, specific activity of Complex IV was followed by polarography as described before ^77,78^ by assessing the KCN-sensitive oxygen uptake rate by using the artificial substrates ascorbate/TMPD (10 mM/100 μM, respectively) in the presence of the Complex III inhibitor antimycin at 4 μM, to evaluate Complex IV only. In separate experiments, various concentrations of NaHS (as a hydrogen sulfide donor) were used to evaluate its effect on Complex IV activity (expressed as nmol oxygen consumed × (min × mg mitochondrial protein)^−1^.

### Supplementary Information

Supplementary Information.

## Data Availability

The images analyzed during the current study are available in the Dryad repository under the title “Electron microscopy images of the thalamus from acutely hydrogen sulfide poisoned mice" and accessible via the following link: https://doi.org/10.5061/dryad.k3j9kd5ds. All data generated and analyzed during this study are included in this published article.
