# Supplementary material for: Disrupted brain mitochondrial morphology after in vivo hydrogen sulfide exposure
Source: Sci Rep. 2023 Oct 24;13:18129. doi: 10.1038/s41598-023-44807-y (PMC10598273; doi:10.1038/s41598-023-44807-y)
Supplement: Supplementary file 1 — Supplementary Information. [file 41598_2023_44807_MOESM1_ESM.pdf]

Supplementary information

Page 1. Mitochondrial area analysis per condition, namely control (breathing air), and at 12, 24, 48, and 72 h post-exposure to hydrogen sulfide. Analysis was performed using JMP software v. 17.2. Data for the area were obtained as detailed under the main text.

Page 2. Mitochondrial density analysis per condition, namely control (breathing air), and at 12, 24, 48, and 72 h post-exposure to hydrogen sulfide. Analysis was performed using JMP software v. 17.2. Data for the density were obtained as detailed under the main text.

Distributions AREA

Control

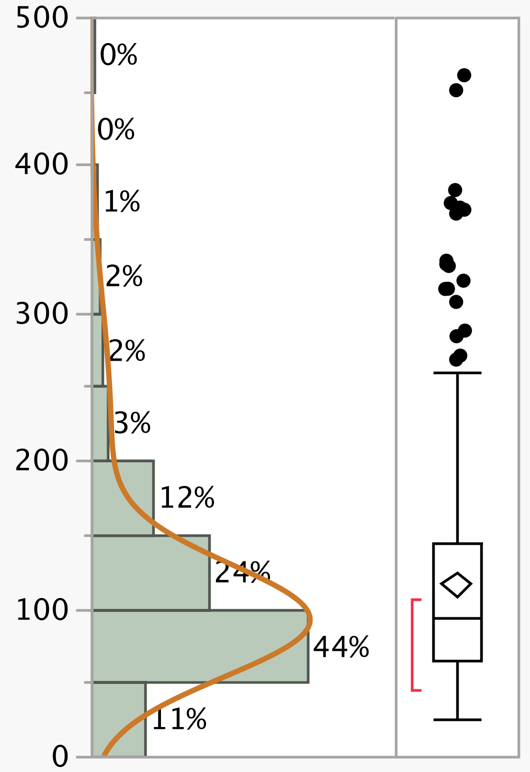

Quantiles

100.0%	maximum	737.89
99.5%		584.1132
97.5%		354.048
90.0%		199.406
75.0%	quartile	144.05
50.0%	median	94.35
25.0%	quartile	64.98
10.0%		48.446
2.5%		33.822
0.5%		27.864
0.0%	minimum	25.14

Summary Statistics

Mean	117.17752
Std Dev	82.813752
Std Err Mean	4.0651672
Upper 95% Mean	125.16846
Lower 95% Mean	109.18658
N	415
N Missing	109

Fitted Normal 3 Mixture Distribution

Parameter		Estimate	Lower 95%	Upper 95%
Location	μ1	91.247318	87.183657	95.310978
Location	μ2	218.38528	198.50391	238.26664
Location	μ3	619.86081	560.43113	679.29049
Dispersion	σ1	38.219823	34.406372	42.455939
Dispersion	σ2	86.17449	69.313415	107.13716
Dispersion	σ3	52.670287	28.549237	97.171041
Probability	π1	0.8191134	0.7155639	0.8907225
Probability	π2	0.1736166	0.1371234	0.2173752
Probability	π3	0.00727	0.0023446	0.0223111

Measures	
-2*LogLikelihood	4577.3766
AICc	4593.7313
BIC	4625.6029

12 hours

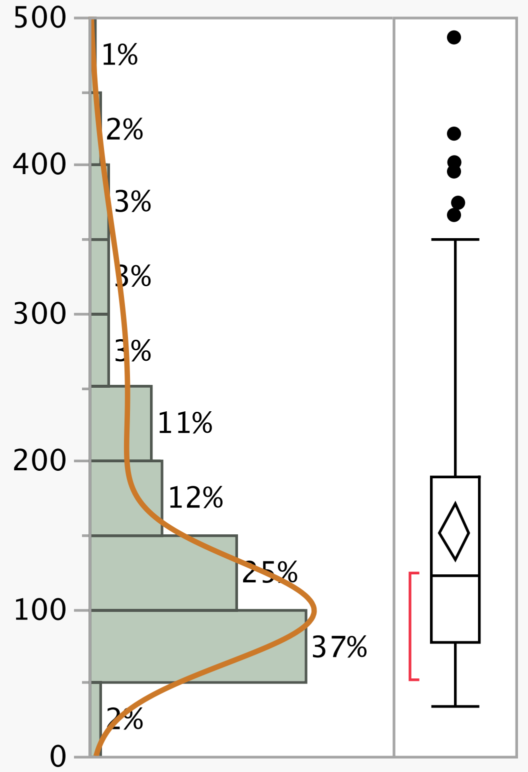

Quantiles

100.0%	maximum	686.55
99.5%		686.55
97.5%		418.933
90.0%		300.902
75.0%	quartile	189.62
50.0%	median	121.89
25.0%	quartile	76.88
10.0%		57.346
2.5%		51.518
0.5%		33.79
0.0%	minimum	33.79

Summary Statistics

Mean	152.11098
Std Dev	105.52539
Std Err Mean	9.5149042
Upper 95% Mean	170.94668
Lower 95% Mean	133.27527
N	123
N Missing	401

Fitted Normal 3 Mixture Distribution

Parameter		Estimate	Lower 95%	Upper 95%
Location	μ1	97.06463	89.333824	104.79544
Location	μ2	245.46064	213.44384	277.47744
Location	μ3	.	.	.
Dispersion	σ1	34.696164	28.044778	42.925061
Dispersion	σ2	110.33763	84.602185	143.90163
Dispersion	σ3	.	.	.
Probability	π1	0.6290788	0.4818982	0.7556492
Probability	π2	0.3709212	0.2710048	0.4832555
Probability	π3	0	.	.

Measures	
-2*LogLikelihood	1430.8439
AICc	1443.5681
BIC	1459.717

24 hours

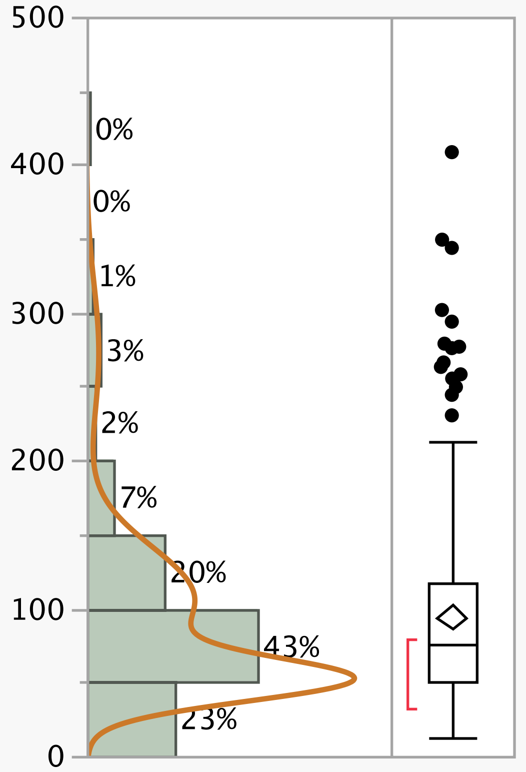

Quantiles

100.0%	maximum	408.35
99.5%		397.102
97.5%		279.5545
90.0%		172.292
75.0%	quartile	116.49
50.0%	median	76.59
25.0%	quartile	51.06
10.0%		38.55
2.5%		28.043
0.5%		14.6009
0.0%	minimum	13.25

Summary Statistics

Mean	94.547046
Std Dev	64.935326
Std Err Mean	4.2180027
Upper 95% Mean	102.85679
Lower 95% Mean	86.237299
N	237
N Missing	287

Fitted Normal 3 Mixture Distribution

Parameter		Estimate	Lower 95%	Upper 95%
Location	μ1	51.111699	48.31662	53.906778
Location	μ2	105.62164	99.161247	112.08203
Location	μ3	272.41043	250.35979	294.46108
Dispersion	σ1	14.399845	11.917084	17.399856
Dispersion	σ2	35.720116	29.996525	42.535818
Dispersion	σ3	47.204853	32.160507	69.286789
Probability	π1	0.4305449	0.3496703	0.5153033
Probability	π2	0.49518	0.406718	0.5839449
Probability	π3	0.074275	0.0462029	0.1173057

Measures	
-2*LogLikelihood	2489.8242
AICc	2506.4558
BIC	2533.5687

48 hours

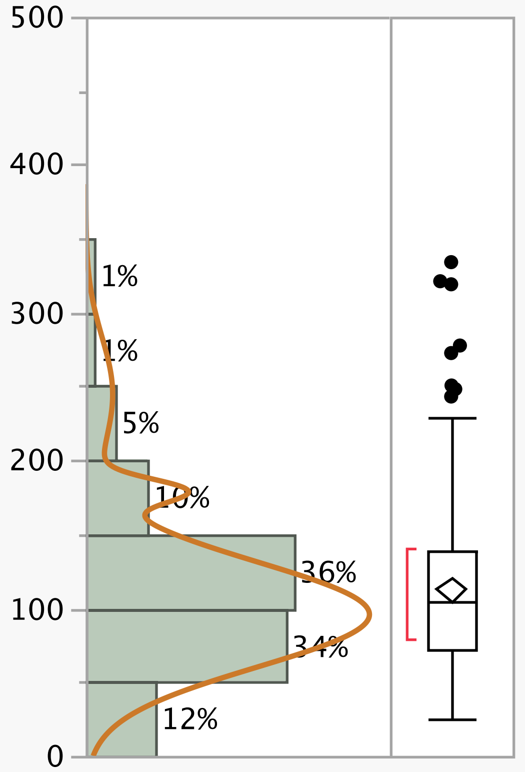

Quantiles

100.0%	maximum	333.95
99.5%		333.7562
97.5%		270.8045
90.0%		186.767
75.0%	quartile	138.72
50.0%	median	104.23
25.0%	quartile	72.6625
10.0%		48.41
2.5%		31.77725
0.5%		25.29425
0.0%	minimum	25.28

Summary Statistics

Mean	113.23698
Std Dev	57.699645
Std Err Mean	4.0597329
Upper 95% Mean	121.24211
Lower 95% Mean	105.23185
N	202
N Missing	322

Fitted Normal 3 Mixture Distribution

Parameter		Estimate	Lower 95%	Upper 95%
Location	μ1	95.584053	90.312966	100.85514
Location	μ2	179.28599	174.52543	184.04654
Location	μ3	244.5986	226.7014	262.49581
Dispersion	σ1	35.405952	30.616775	40.944266
Dispersion	σ2	8.0064081	4.3383873	14.775668
Dispersion	σ3	38.541076	26.293305	56.494022
Probability	π1	0.8580191	0.6792598	0.9451891
Probability	π2	0.0537905	0.029433	0.0963049
Probability	π3	0.0881905	0.0549293	0.1386376

Measures	
-2*LogLikelihood	2165.3576
AICc	2182.1037
BIC	2207.8238

72 hours

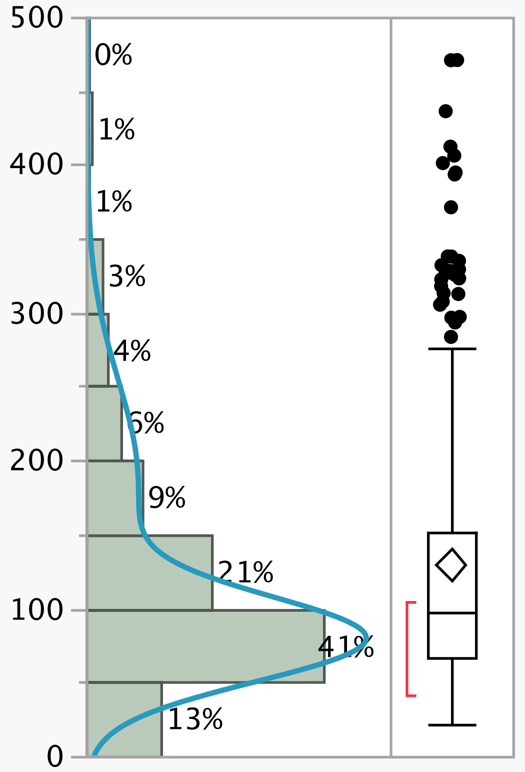

Quantiles

100.0%	maximum	1432.428
99.5%		944.03863
97.5%		411.31013
90.0%		245.755
75.0%	quartile	150.998
50.0%	median	96.671
25.0%	quartile	66.5525
10.0%		45.9145
2.5%		32.63875
0.5%		23.68275
0.0%	minimum	21.25

Summary Statistics

Mean	130.24321
Std Dev	122.29716
Std Err Mean	5.342576
Upper 95% Mean	140.73876
Lower 95% Mean	119.74767
N	524
N Missing	0

Fitted Normal 3 Mixture Distribution

Parameter		Estimate	Lower 95%	Upper 95%
Location	μ1	78.559573	75.497634	81.621512
Location	μ2	183.08445	172.18715	193.98174
Location	μ3	514.17564	405.42446	622.92683
Dispersion	σ1	28.655079	25.77495	31.857037
Dispersion	σ2	71.416164	61.542137	82.874414
Dispersion	σ3	263.62302	185.92371	373.79363
Probability	π1	0.6420622	0.5709667	0.7074136
Probability	π2	0.3148594	0.2689003	0.3647547
Probability	π3	0.0430784	0.028421	0.064791

Measures	
-2*LogLikelihood	5906.3532
AICc	5922.6328
BIC	5956.4452

Distributions DENSITY

Control

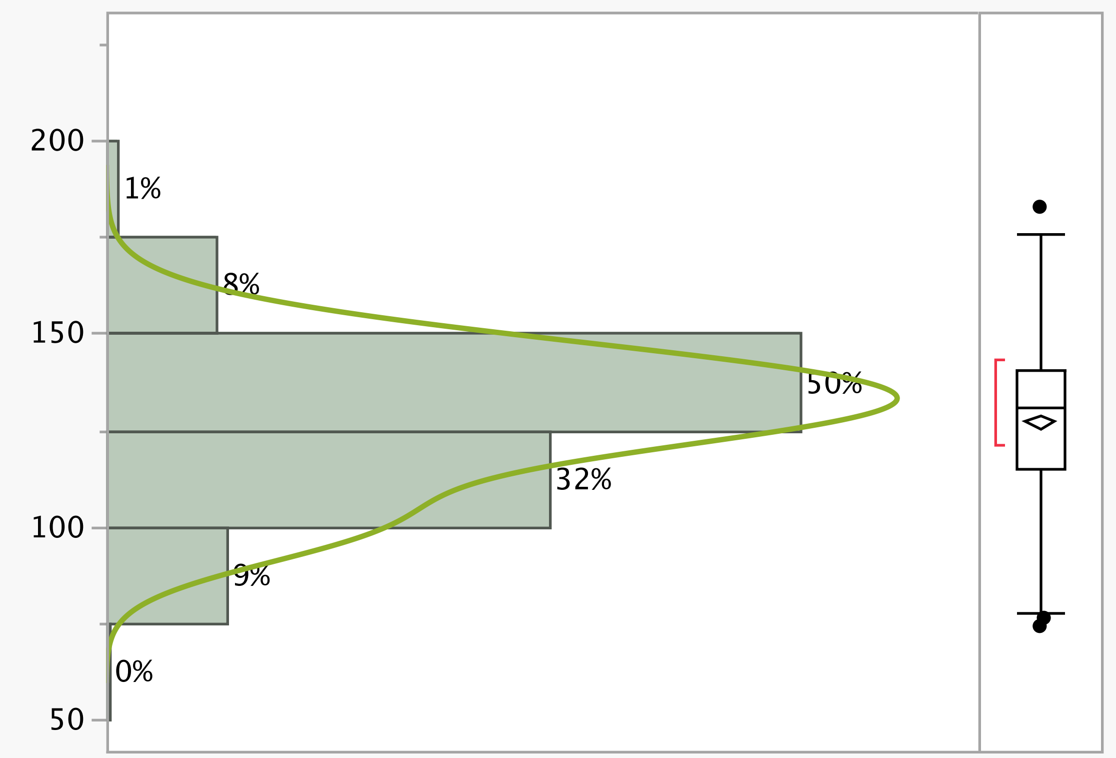

Quantiles

100.0%	maximum	182.9
99.5%		175.9696
97.5%		161.562
90.0%		147.638
75.0%	quartile	140.29
50.0%	median	130.64
25.0%	quartile	114.76
10.0%		101.044
2.5%		87.29
0.5%		76.0848
0.0%	minimum	73.81

Summary Statistics

Mean	127.31231
Std Dev	18.87923
Std Err Mean	0.9267449
Upper 95% Mean	129.13403
Lower 95% Mean	125.4906
N	415
N Missing	109

Fitted Normal 2 Mixture Distribution

Parameter		Estimate	Lower 95%	Upper 95%
Location	μ1	99.771294	97.35022	102.19237
Location	μ2	133.20069	131.67995	134.72143
Dispersion	σ1	10.560712	8.4916763	13.133877
Dispersion	σ2	14.347024	12.920529	15.931011
Probability	π1	0.1761281	0.1393059	0.2201932
Probability	π2	0.8238719	0.7192989	0.895165

Measures

-2*LogLikelihood	3600.7643
AICc	3610.911
BIC	3630.9057

12 hours

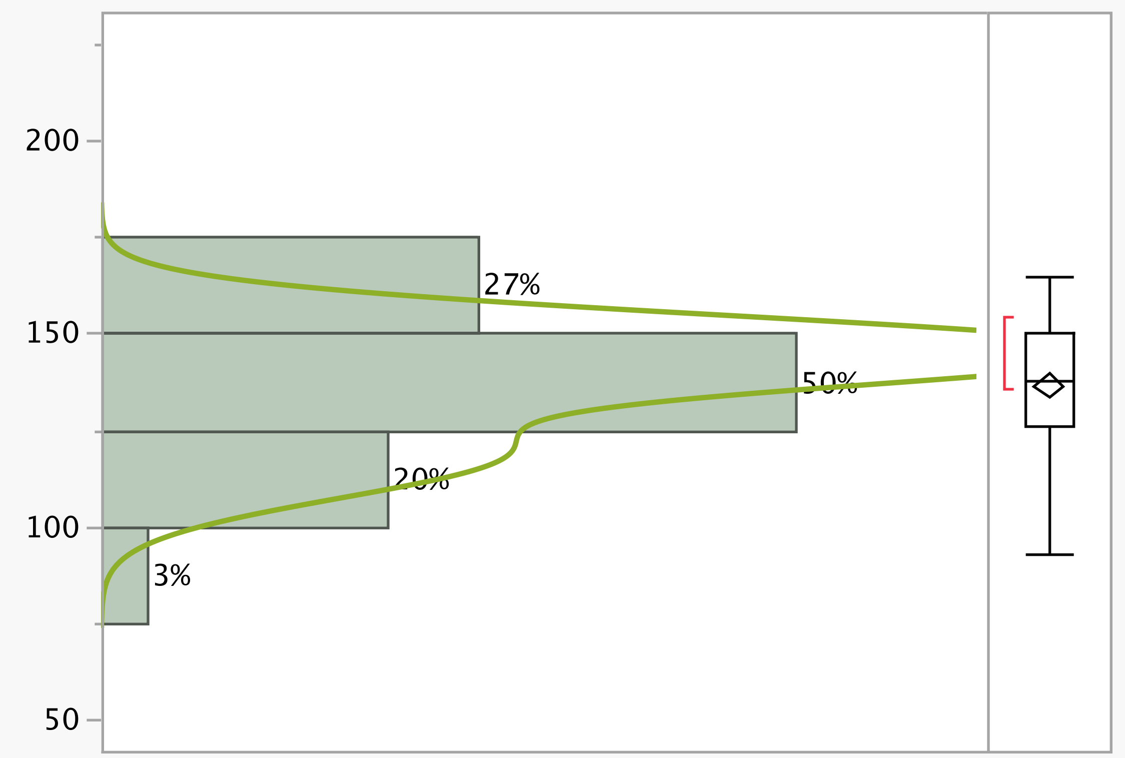

Quantiles

100.0%	maximum	165.26
99.5%		165.26
97.5%		162.45
90.0%		155.578
75.0%	quartile	150.68
50.0%	median	137.9
25.0%	quartile	126.3
10.0%		116.552
2.5%		98.455
0.5%		92.51
0.0%	minimum	92.51

Summary Statistics

Mean	136.80496
Std Dev	16.365112
Std Err Mean	1.4755925
Upper 95% Mean	139.72604
Lower 95% Mean	133.88388
N	123
N Missing	401

Fitted Normal 2 Mixture Distribution

Parameter		Estimate	Lower 95%	Upper 95%
Location	μ1	118.33028	114.79467	121.86589
Location	μ2	145.34329	143.365	147.32157
Dispersion	σ1	11.247313	8.4518178	14.967437
Dispersion	σ2	9.2577234	7.546338	11.357223
Probability	π1	0.3160522	0.2259037	0.4225385
Probability	π2	0.6839478	0.5239445	0.8097054

Measures

-2*LogLikelihood	1023.9083
AICc	1034.4211
BIC	1047.9692

24 hours

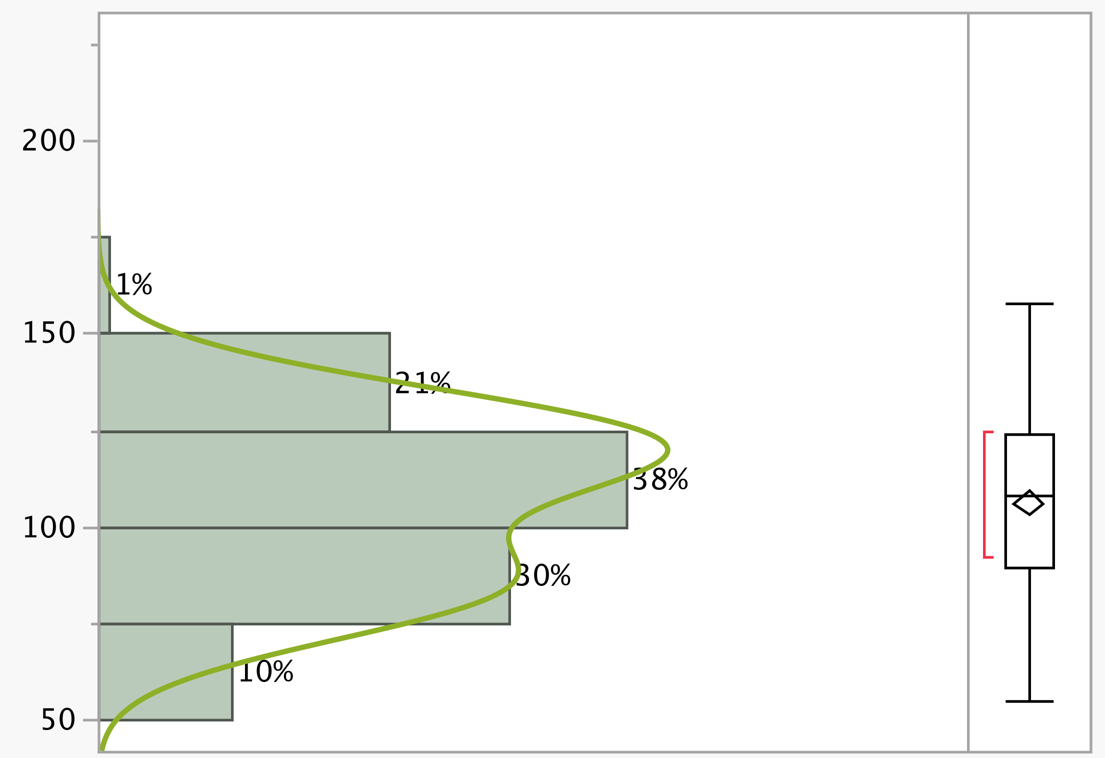

Quantiles

100.0%	maximum	157.73
99.5%		156.4057
97.5%		145.7
90.0%		138.222
75.0%	quartile	124.005
50.0%	median	107.83
25.0%	quartile	89.365
10.0%		75.25
2.5%		57.2405
0.5%		54.3013
0.0%	minimum	54.25

Summary Statistics

Mean	106.23342
Std Dev	23.343111
Std Err Mean	1.516298
Upper 95% Mean	109.22063
Lower 95% Mean	103.24621
N	237
N Missing	287

Fitted Normal 2 Mixture Distribution

Parameter		Estimate	Lower 95%	Upper 95%
Location	μ1	84.326699	81.464469	87.188928
Location	μ2	120.85944	118.42373	123.29514
Dispersion	σ1	14.213059	11.710545	17.250353
Dispersion	σ2	14.823185	12.635175	17.39009
Probability	π1	0.4003453	0.3230362	0.4829568
Probability	π2	0.5996547	0.498397	0.6930602

Measures

-2*LogLikelihood	2153.9288
AICc	2164.1885
BIC	2181.2691

48 hours

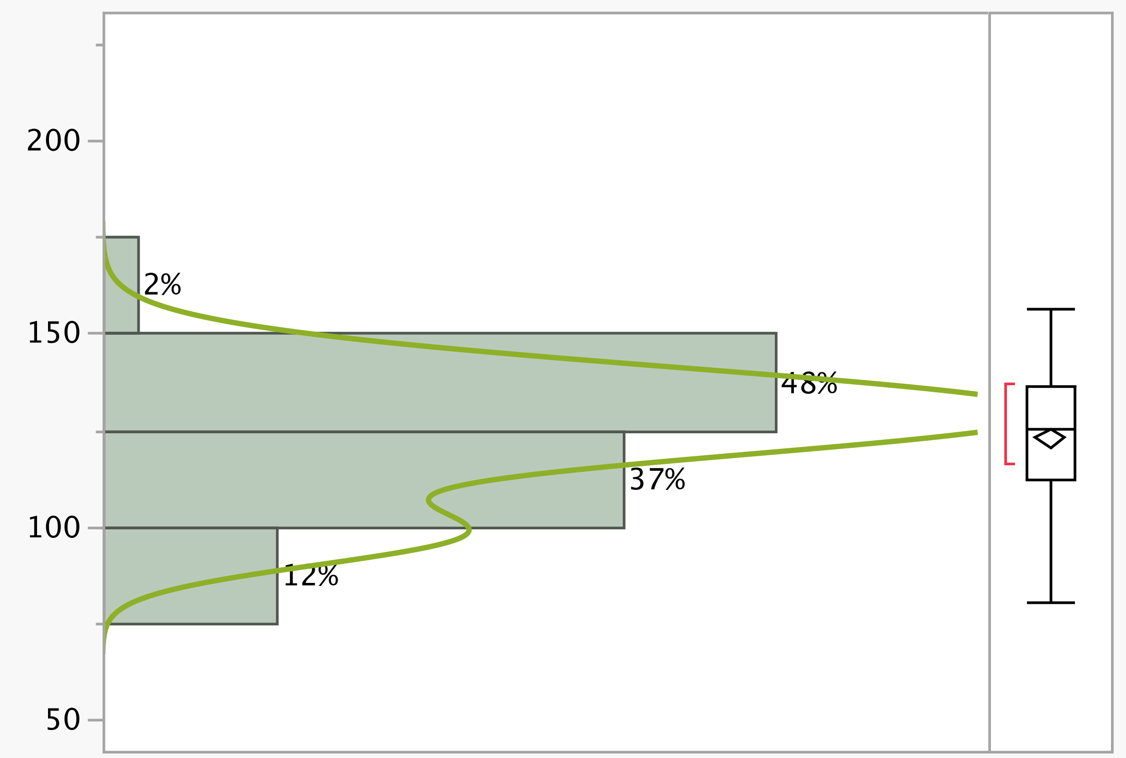

Quantiles

100.0%	maximum	156.65
99.5%		156.60455
97.5%		150.404
90.0%		143.571
75.0%	quartile	136.3225
50.0%	median	125.075
25.0%	quartile	112.1525
10.0%		97.953
2.5%		86.656
0.5%		79.92245
0.0%	minimum	79.91

Summary Statistics

Mean	123.10223
Std Dev	16.987966
Std Err Mean	1.1952691
Upper 95% Mean	125.4591
Lower 95% Mean	120.74535
N	202
N Missing	322

Fitted Normal 2 Mixture Distribution

Parameter		Estimate	Lower 95%	Upper 95%
Location	μ1	97.373977	94.872038	99.875916
Location	μ2	129.17771	127.36166	130.99376
Dispersion	σ1	7.9290247	5.9336276	10.595446
Dispersion	σ2	11.844864	10.199055	13.756254
Probability	π1	0.1909998	0.137814	0.258556
Probability	π2	0.8090002	0.6549354	0.9043264

Measures

-2*LogLikelihood	1700.4568
AICc	1710.7629
BIC	1726.9981

72 hours

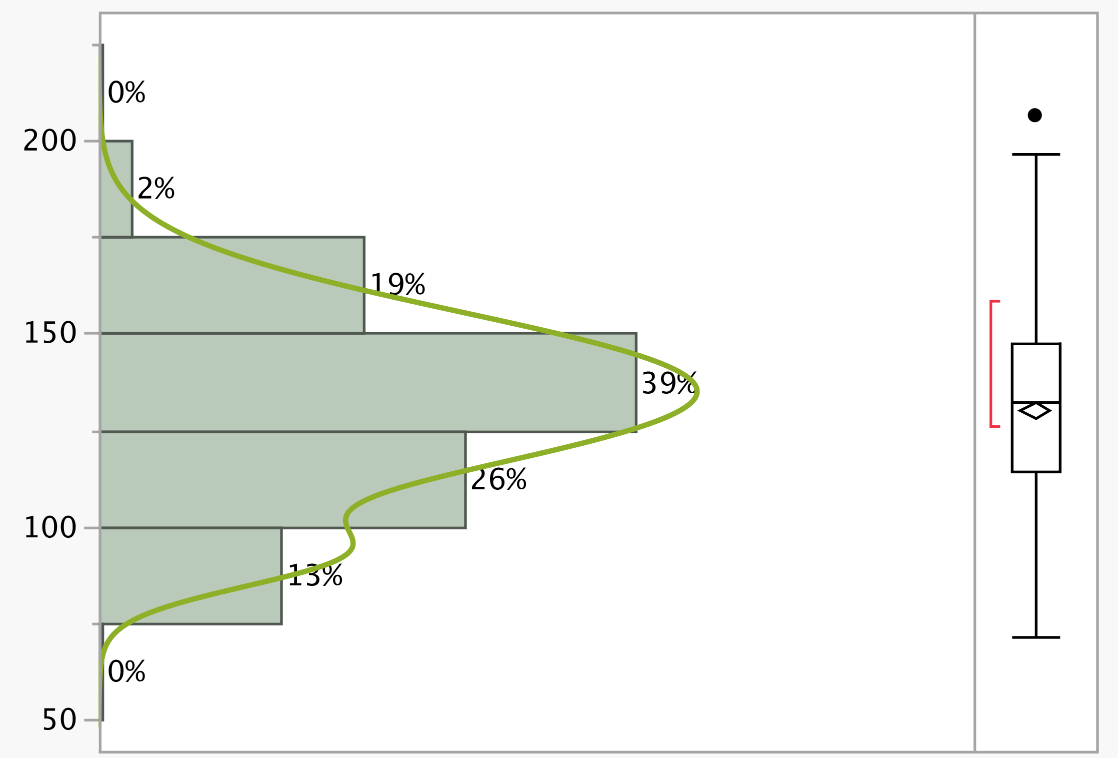

Quantiles

100.0%	maximum	206.716
99.5%		194.51263
97.5%		175.028
90.0%		158.69
75.0%	quartile	147.3825
50.0%	median	132.474
25.0%	quartile	114.0475
10.0%		96.376
2.5%		82.178875
0.5%		76.41625
0.0%	minimum	70.855

Summary Statistics

Mean	130.29801
Std Dev	23.879735
Std Err Mean	1.0431911
Upper 95% Mean	132.34737
Lower 95% Mean	128.24865
N	524
N Missing	0

Fitted Normal 2 Mixture Distribution

Parameter		Estimate	Lower 95%	Upper 95%
Location	μ1	91.567049	89.397008	93.73709
Location	μ2	134.85694	132.99058	136.72329
Dispersion	σ1	8.2235691	6.3940224	10.576611
Dispersion	σ2	20.618408	18.849331	22.553519
Probability	π1	0.1052965	0.0805684	0.1364874
Probability	π2	0.8947035	0.7824592	0.9525456

Measures

-2*LogLikelihood	4794.1428
AICc	4804.2586
BIC	4825.4502
